# Supplementary material for: In Silico and Cellular Differences Related to the Cell Division Process between the A and B Races of the Colonial Microalga Botryococcus braunii
Source: Biomolecules. 2021 Oct 5;11(10):1463. doi: 10.3390/biom11101463 (PMC8533097; doi:10.3390/biom11101463)
Supplement: Supplementary file 1 [file biomolecules-11-01463-s001.zip › biomolecules-1394059-supplementary.pdf]

Supplementary Materials

Table S1. Comparison of E-values and % of identity of MAT3/RBR/RBR1 and CDKs sequences.

**MAT3/RBR - *B. braunii* race A (Yamanaka)**	**MAT3/RBR/RBR1**	**Max Score**	**Total Score**	**Query Cover**	**E value**	**% ident.**	**Accession**
G. pectorale	MAT3/RBR	266	266	45%	5.00E-74	35.02	BAN18532.1
V. carteri f. *nagariensis* (m)	MAT3/RBR	254	254	60%	2.00E-69	31.7	ADI46925.1
V. carteri f. *nagariensis* (f)	MAT3/RBR	263	263	65%	9.00E-73	30.28	ABM47317.1
O. tauri	MAT3/RBR	204	204	54%	3.00E-53	29.58	OUS45688.1
A. thaliana	RBR1	229	229	60%	2.00E-61	29.23	NP_566417.3
C. reinhardtii	MAT3/RBR	144	286	64%	7.00E-34	27.75	XP_001696629.1
CDKA1 - *B. braunii* race A (Yamanaka)	CDKs	Max Score	Total Score	Query Cover	E value	% ident.	Accession
A. thaliana	CDKA;1	454	454	85%	2.00E-161	72.76	NP_566911.1
V. carteri f. *nagariensis*	CDKA1	440	440	85%	1.00E-155	71.72	XP_002949867.1
C. reinhardtii	CDKA1	446	446	85%	2.00E-157	71.03	XP_001698637.1
G. pectorale	CDKA1	437	437	85%	2.00E-154	70.24	KXZ46110.1
O. tauri	CDKA1	420	420	85%	7.00E-148	67.82	XP_003078530.1
CDKB1 - *B. braunii* race A (Yamanaka)	CDKs	Max Score	Total Score	Query Cover	E value	% ident.	Accession
G. pectorale	CDKB1	514	514	97%	0	78.57	KXZ43845.1
V. carteri f. *nagariensis*	CDKB1	524	524	99%	0	78.34	XP_002947156.1
C. reinhardtii	CDKB1	517	517	99%	0	77.71	XP_001701299.1
O. tauri	CDKB1	473	473	97%	2.00E-168	72.17	XP_003083211.1
A. thaliana	CDKB1;1	445	445	94%	9.00E-158	68.73	NP_190986.1
A. thaliana	CDKB1;2	449	449	94%	2.00E-159	68.61	NP_001031507.1
A. thaliana	CDKB2;1	399	399	94%	1.00E-139	61.92	NP_177780.1
A. thaliana	CDKB2;2	396	396	95%	2.00E-138	60.98	NP_173517.1
CDKD1 - *B. braunii* race A (Yamanaka)	CDKs	Max Score	Total Score	Query Cover	E value	% ident.	Accession
A. thaliana	CDKD;2	398	398	75%	3.00E-137	60.07	NP_176847.1
A. thaliana	CDKD;1	417	417	96%	3.00E-144	54.22	NP_177510.1
C. reinhardtii	CDKD1	324	324	78%	3.00E-108	52.65	XP_001694537.1
A. thaliana	CDKD;3	415	415	96%	3.00E-143	52.56	NP_173244.1
O. tauri	CDKD1	374	374	85%	5.00E-127	52.34	AAV68598.1
G. pectorale	CDKD1	318	318	76%	6.00E-99	52.24	KXZ54250.1
V. carteri f. *nagariensis*	CDKD1	333	333	85%	1.00E-111	50.56	XP_002954735.1
CDKE1 - *B. braunii* race A (Yamanaka)	CDKs	Max Score	Total Score	Query Cover	E value	% ident	Accession
C. reinhardtii	CDKE1	425	425	74%	2.00E-144	59.46	PNW83964.1
A. thaliana	CDKE;1	422	422	74%	9.00E-144	58.31	NP_201166.1
G. pectorale	CDKE1	376	376	69%	3.00E-127	58.05	KXZ45461.1
V. carteri f. *nagariensis*	CDKE1	409	409	76%	3.00E-139	57.87	XP_002957533.1
O. tauri	CDKE1	251	251	83%	6.00E-78	40.1	XP_022840036.1
CDKE2 - *B. braunii* race A (Yamanaka)	CDKs	Max Score	Total Score	Query Cover	E value	% ident	Accession
C. reinhardtii	CDKE1	403	403	85%	2.00E-137	60.06	PNW83964.1
A. thaliana	CDKE;1	403	403	84%	1.00E-137	59.57	NP_201166.1
G. pectorale	CDKE1	359	359	82%	3.00E-122	58.16	KXZ45461.1
V. carteri f. *nagariensis*	CDKE1	391	391	91%	6.00E-134	57.69	XP_002957533.1

O. tauri	CDKE1	244	244	64%	2.00E-76	51.32	XP_022840036.1
CDKG1 - *B. braunii* race A (Yamanaka)	CDKs	Max Score	Total Score	Query Cover	E value	% ident	Accession
O. tauri	CDKG2/CDK10	405	405	92%	2.00E-140	54.91	XP_003080520.2
A. thaliana	CDKG;2	372	372	88%	2.00E-122	53.87	NP_001154456.1
A. thaliana	CDKG;1	348	348	82%	9.00E-115	51.09	OAO92015.1
O. tauri	CDKG1	325	325	81%	9.00E-106	49.84	XP_003074327.1
V. carteri f. *nagariensis*	CDKG1	233	233	77%	2.00E-72	42.32	XP_002946192.1
C. reinhardtii	CDKG1	225	225	77%	2.00E-69	42.32	XP_001696492.1
G. pectorale	CDKG1	217	217	77%	3.00E-66	40.61	KXZ52035.1
C. reinhardtii	CDKG2	197	197	76%	2.00E-59	38.28	XP_001701126.1
CDKH1 - *B. braunii* race A (Yamanaka)	CDKs	Max Score	Total Score	Query Cover	E value	% ident	Accession
V. carteri f. *nagariensis*	CDKH1	329	329	41%	2.00E-106	54.26	XP_002956993.1
C. reinhardtii	CDKH1	377	377	47%	4.00E-123	53.2	XP_001702056.1
G. pectorale	CDKH1	347	347	44%	9.00E-112	52.37	KXZ53820.1
CDKH2 - *B. braunii* race A (Yamanaka)	CDKs	Max Score	Total Score	Query Cover	E value	% ident	Accession
V. carteri f. *nagariensis*	CDKH1	332	332	39%	6.00E-108	57.73	XP_002956993.1
G. pectorale	CDKH1	355	355	42%	1.00E-115	56.73	KXZ53820.1
C. reinhardtii	CDKH1	386	386	45%	9.00E-127	56.59	XP_001702056.1
CDKI1 - *B. braunii* race A (Yamanaka)	CDKs	Max Score	Total Score	Query Cover	E value	% Ident	Accession
C. reinhardtii	CDKI1	151	151	68%	2.00E-42	43.1	XP_001700559.1
V. carteri f. nagariensis	CDKI1	194	194	83%	6.00E-58	42.76	XP_002956880.1
G. pectorale	CDKI1	179	179	86%	1.00E-51	38.12	KXZ49794.1
**MAT3/RBR - *B. braunii* race B (Showa)**	**MAT3/RBR/RBR1**	**Max Score**	**Total Score**	**Query Cover**	**E value**	**% ident**	**Accession**
G. pectorale	MAT3/RBR	290	290	77%	7.00E-82	29.67	BAN18532.1
V. carteri f. *nagariensis* (f)	MAT3/RBR	283	283	78%	3.00E-79	29.02	ABM47317.1
V. carteri f. *nagariensis* (m)	MAT3/RBR	268	268	75%	5.00E-74	28.08	ADI46925.1
O. tauri	MAT3/RBR	245	245	81%	8.00E-67	27.02	OUS45688.1
A. thaliana	RBR1	251	251	84%	1.00E-68	26.87	NP_566417.3
C. reinhardtii	MAT3/RBR	159	304	75%	1.00E-38	26.74	XP_001696629.1
CDKA1 - *B. braunii* race B (Showa)	CDKs	Max Score	Total Score	Query Cover	E value	% ident	Accession
V. carteri f. *nagariensis*	CDKA1	445	445	90%	8.00E-158	71.48	XP_002949867.1
A. thaliana	CDKA;1	449	449	90%	2.00E-159	71.38	NP_566911.1
G. pectorale	CDKA1	447	447	91%	8.00E-159	70.75	KXZ46110.1
C. reinhardtii	CDKA1	448	448	90%	1.00E-158	70.45	XP_001698637.1
O. tauri	CDKA1	414	414	90%	6.00E-146	64.95	XP_003078530.1
CDKB1 - *B. braunii* race B (Showa)	CDKs	Max Score	Total Score	Query Cover	E value	% ident	Accession
G. pectorale	CDKB1	516	516	97%	0	79.22	KXZ43845.1
V. carteri f. *nagariensis*	CDKB1	521	521	99%	0	78.66	XP_002947156.1
C. reinhardtii	CDKB1	515	515	99%	0	78.03	XP_001701299.1
O. tauri	CDKB1	469	469	97%	5.00E-167	71.52	XP_003083211.1
A. thaliana	CDKB1;1	444	444	94%	1.00E-157	69.28	NP_190986.1
A. thaliana	CDKB1;2	445	445	94%	7.00E-158	67.53	NP_001031507.1
A. thaliana	CDKB2;1	400	400	95%	3.00E-140	62.42	NP_177780.1
A. thaliana	CDKB2;2	395	395	95%	3.00E-138	61.76	NP_173517.1
CDKC1 - *B. braunii* race B (Showa)	CDKs	Max Score	Total Score	Query Cover	E value	% ident	Accession

O. tauri	CDKC1	414	414	54%	1.00E-137	60.29	AAV68597.1
C. reinhardtii	CDKC1	411	411	56%	2.00E-137	57.66	XP_001694199.1
V. carteri f. *nagariensis*	CDKC1	423	423	57%	2.00E-141	57.22	XP_002954450.1
G. pectorale	CDKC1	394	394	61%	8.00E-130	50.95	KXZ43562.1
A. thaliana	CDKC;2	419	419	71%	8.00E-140	50.74	NP_201301.1
A. thaliana	CDKC;1	423	423	79%	9.00E-142	49.2	NP_196589.1
CDKD1 - *B. braunii* race B (Showa)	CDKs	Max Score	Total Score	Query Cover	E value	% ident	Accession
A. thaliana	CDKD;3	399	399	76%	4.00E-137	60.4	NP_173244.1
A. thaliana	CDKD;1	398	398	77%	7.00E-137	60.4	NP_177510.1
A. thaliana	CDKD;2	379	379	77%	6.00E-130	56.58	NP_176847.1
O. tauri	CDKD1	390	390	83%	6.00E-134	55.86	AAV68598.1
C. reinhardtii	CDKD1	327	327	72%	1.00E-109	55.36	XP_001694537.1
G. pectorale	CDKD1	325	325	78%	1.00E-101	53.53	KXZ54250.1
V. carteri	CDKD1	333	333	84%	6.00E-112	52.37	XP_002954735.1
CDKE1 - *B. braunii* race B (Showa)	CDKs	Max Score	Total Score	Query Cover	E value	% ident	Accession
V. carteri f. *nagariensis*	CDKE1	398	398	72%	3.00E-135	58.72	XP_002957533.1
A. thaliana	CDKE;1	414	414	74%	9.00E-141	57.65	NP_201166.1
C. reinhardtii	CDKE1	408	408	75%	3.00E-138	57.02	PNW83964.1
G. pectorale	CDKE1	365	365	72%	2.00E-123	55.46	KXZ45461.1
O. tauri	CDKE1	252	252	76%	9.00E-79	43.01	XP_022840036.1
CDKG1 - *B. braunii* race B (Showa)	CDKs	Max Score	Total Score	Query Cover	E value	% ident	Accession
O. tauri	CDKG2/CDK10	404	404	92%	6.00E-140	54.31	XP_003080520.2
A. thaliana	CDKG;2	365	365	84%	1.00E-119	54.15	NP_001154456.1
A. thaliana	CDKG;1	343	343	81%	1.00E-112	50.94	OAO92015.1
O. tauri	CDKG1	316	316	79%	2.00E-102	49.35	XP_003074327.1
V. carteri f. *nagariensis*	CDKG1	237	237	77%	3.00E-74	42.32	XP_002946192.1
C. reinhardtii	CDKG1	222	222	77%	2.00E-68	40.27	XP_001696492.1
G. pectorale	CDKG1	220	220	78%	2.00E-67	39.66	KXZ52035.1
C. reinhardtii	CDKG2	197	197	76%	2.00E-59	37.37	XP_001701126.1
CDKH1 - *B. braunii* race B (Showa)	CDKs	Max Score	Total Score	Query Cover	E value	% ident	Accession
G. pectorale	CDKH1	377	377	47%	2.00E-124	57.59	KXZ53820.1
C. reinhardtii	CDKH1	407	407	49%	2.00E-135	57.52	XP_001702056.1
V. carteri f. *nagariensis*	CDKH1	343	343	47%	1.00E-112	54.49	XP_002956993.1
CDKI1 - *B. braunii* race B (Showa)	CDKs	Max Score	Total Score	Query Cover	E value	% ident	Accession
V. carteri f. nagariensis	CDKI1	209	209	78%	6.00E-63	40.87	XP_002956880.1
C. reinhardtii	CDKI1	159	159	77%	1.00E-44	37.23	XP_001700559.1
G. pectorale	CDKI1	189	189	77%	1.00E-54	36.49	KXZ49794.1

Sequences were from [54].

Table S2 Amino acid sequences of RBR and CDKs from *B. braunii*.

**MAT3/RBR Sequence**
**>Bbra:MAT3/RBR from race A**
MAEVGPAEFDRLILGCGIEFEPELLDAARSVVEKCNGDVCKRYGHASMPSEHLQAWSRLWFASVLFFTAKL<br>RASQRAGSPDSTHRSTPTLSQILVAFDVSLAAFMKPALLALFGNEPPLERRLQLRELQVNYVYSAVMAKKYR<br>DFFTKVCTLPVEGDGSEVFKLFWTLFLVAKASLLEPFAAEELNPPSSDCLASSCVRVEGLLSDAEVTVSITNTL<br>NAHYEQVYRAGQVDERPLILAQACEQASEPANGPGPLPAPSSGAATPSMSKCNVSTPLQNRQLTAFSPAIQ<br>PSSMNSVLVPMLTGTPMRSMMSPIEGVNGCRMLLTPSPARGPHNFLVPPQHIMETPVTEAMAAVHWLLH<br>QNMSETQAPEPSMELQRFFSECGEGPGEAIAEGVEQMANLVFPESPQNSQDEVGVSSISMPNRRQCGIRLYY<br>LVLDAMRKGDQEKPGRASFATTIKSPSFHKCMLACAFELVIASYRMVTHTFPTVLERLHLKPFDLCKLISTFV<br>RHLPNLPRELKRHMFSIEEKCLESLSWERGSSLYPLLQTACDPNPSPPDNVMVDAEQPVSPQERKARSPKKM<br>DLTPTKRGREESPQHFENGHAAMRNGDATLAEAPAHANGAMPGSAPSDMAVDTTAAAPLPLAFGRPGG<br>SNAERPGRQVVADFLQRVLRLAYLRITDLCERLDFEPLERQVLVLEVYTMLHYCVYEVTSLLYGRHLDQLLL<br>CALYGVCKVHQLRQITFKDIIAQYKRQPQCLTNTFRSVAIELTPGLQVLETGDVIMFYNKKFIPATKQFVLAL<br>GQRDVPIIKPPVIGPTLSSRQATPQNSAGAGLASPPPSAVRNGGSLLVSPLRRNRNQFGWGQSSWTGVKPFT<br>VELGRSPRRSVDFVNTILHNREYRGHPLFSAIGGMPGGAGSSDHSHGSIDSEAHHTYVKPVVRDIFGSGHIPL<br>RMRSSGQGGVDESDEDARGSPIVANGGLPPARDMKRQCAVFP
**CDKA1 Sequence**
**>Bbra:CDKA1 from race A**
MGTLQGMDKYEKLDKIGEGTYGVVYKARNKMTGEIIALKKIRLEQEEEGVPSTAIREISLLKELTHINVVRLH<br>DVIHSEKRLHLVFEYCDLDLKKHMDQNPVLCKDRRIIKLYLYQMLQGIAYCHSHRVLHRDLKPQNLLIDRA<br>TNAVKLADFGLARAFGLPTRAYTHEVVTLWYRAPEILLGAKHYSTPVDVWSIGCIFAEMVTQRPLFPGDSEI<br>DQLFKIFRSLGTPDDTVWPGVRELPDYKDTFPKWQRRPLNEILPDLEERGIDLLNQMLRYSPSQRITAKAAL<br>AHPYFADIHMLVNQSRVWIGPNNQAYPASNQAYPASMRGMTHYMQPRPTH
**CDKB1 Sequence**
**>Bbra:CDKB1 from race A**
MDQYEKLEKIGEGTYGKVYKARDIQTGKLVALKKTRLEMEQEGVPSTALREVSLLQMLSESNHVVKLLCVE<br>HVEEQNKPVLYLVFEFLSTDLKKYMDRTGKGPSSAPLPANLVKSFMYQLIKGVAHCHKHGVMHRDLKPQ<br>NLLVDDTKQCLKIADLGLGRAFSIPIKSYTHEIVTLWYRAPEVLLGCTHYSPAVDMWSVACIFAELVRKQAL<br>FPGDSELQQLLHIFKLLGTPDETTWPGVTKLRDWHEFPHWRPQDFKKVFPTLSSEGIDLMAAMFIYDPACRI<br>TAKEALQHPYFDDLDKAAVDLLESDIIRARECS
**CDKD1 Sequence**
**>Bbra:CDKD1 from race A**
MENYIKGETLGEGTFGIVFKACHKETGQTVAIKKIRLGKAKEGVNVTALREIKLLRELDSPYIVRLLDVFPHK<br>QNLNLVFEFMESDLEALIRDRSIIISPPDIKAYMRMGLQALDFCHSRWVLHRDVKPNNFLMSATGEMKLAD<br>FGLSRVFASPDPQYTNQVFSRWYRAPELLFGSTLYGPGVDIWGLGCVFAELLLRRPWLPGNSDIDQLSRIFQA<br>LGTPTTAQWPRMRDLPQFVEFQPSVAPPLRSTFRQASEDALDLLSRMVAYDPARRITAAEALKHRYFRSEPA<br>ATPPARLPRPPIKAHHPLATSTMGGSAIQQAPGEGISQEGQDANGAAAPDAVDGLPDAGHANGGSAPPST<br>GGHTTNGGGTPSRPQCKDEDLRYLRKRKLFSEDALQTPGTSAT
**CDKE1 Sequence**
**>Bbra:CDKE1 from race A**
MEHARSQPGYDQQQGDASTQGAPPQGMPPSSPPPAASHHSPTYLDGFFDMVGKIGEGTYGVVYLARSRET<br>HPRLLAIKTFKPGKEGDGISPTAIREIMLLREIDHQNIVRLDSVHLNRADPCLSLAFDYAEHDLYEMIRHHRD<br>KSHGGLALDPYTLKSVMWQLLNGLSYLHQNWIMHRDLKPSNVLVMGEGGETGKVKIGDFGLARIFKDPLR<br>PLSDNGVVVTIWYRAPELLLGAKHYTKAIDMWAVGCIFAELLLLRPLFQGDEKKHPNNAFQTDQLERIFRL<br>MGPPNARHWPALEQLQHWRDNTENVRVRRPEHPASGPRLAEHLVEHTAMSAGGSCAQGLQPGCAAIDI<br>LTRMLDYNPDTRLTAIQALEHPYFSEEPLPGPNAFVNAGRTVASYPKRAKYSATAANVPIHAQQNSAYENP<br>GAPAQSVSRNATPILGSRAPVPAGNPALVRGVPPGHPSGLPRKRKMDQLGPGFR
**CDKE2 Sequence**

>Bbra:CDKE2 from race A
MEHARSQPGYDQQQGDASTQGAPPQGMPPSSPPPAASHHSPTYLDGFFDMVGKIGEGTYGVVYLARSRET<br>HPRLLAIKTFKPGKEGDGISPTAIREIMLLREIDHQNIVRLDSVHLNRADPCLSLAFDYAEHDLYEMIRHHRD<br>KSHGGLALDPYTLKSVMWQLLNGLSYLHQNWIMHRDLKPSNVLVMGEGGETGKVKIGDFGLARIFKDPLR<br>PLSDNGVVVTIWYRAPELLLGAKHYTKAIDMWAVGCIFAELLLLRPLFQGDEKKHPNNAFQTDQLERIFRL<br>MGPPNARHWPALEQLQHWRDNTENVRVRRPEHPASGPRLAEHLVEHTAMSAGGSCAQGLQPGCAAIDI<br>LTRMLDYNPDTRLTAIQALEHPYFSEVSPIEICWSMP
**CDKG1 Sequence**
**>Bbra:CDKG1 from race A**
MQPDSLPVMASVTKYEKIARVGEGTYGVVYKARDRETGEVVALKKVRMEKERDGMPVTAMREMRVLQTC<br>RHPNLVALKEVVTGSKPTSIFLVFEYCAHDMGRLLDGMPQPFTISEVKCLMTQLLEAVAYLHSRWVMHRD<br>LKLSNLLLTGSGHLKLCDFGLARSFHSFEQAYTPRVITLWYRAPEILLGVEEYTEAVDMWSLGAILGELLNHE<br>PLFPGKSEADMLDMMCSMLGTPSDAIWPGYSKLPNSSKLRLPAQPYNFLAKTFPKVSEAGIDLLNRLLTYHP<br>ESRITARAALRHPFFREVPRAKAPADMPTFPSDRDLPAGGRRHARRVEADAEERGMKRGRGADLDSRFGE<br>AFADGSAHMRARHVRRRP
**CDKH1 Sequence**
**>Bbra:CDKH1 from race A**
MGGSLKRGEKDHPRSSSHDQRRDEGRSERAASAGAGQRRHRPEDEGRRPDGADAHARSHHADRGAYRV<br>PHASSGRRPSGSSRLGGPTHSEPGPLEEAGRQPSARPPRSPYGGDSLLSELRRAAAQTLKRDQEGKERGMSG<br>TRQDARGSDSDGEGQDDEEGELPMNRQEPAPGTGGTRVGSAKRRHADEGGEVTKERGREGLPPKDVKRV<br>RMASPEVADARHHGRQHERGGDAARGKGAAAIHTAGSSSRLAAVSPLVSRSAVEIAQQQLELFEQQAQL<br>MEDIDPDAPAAMKPSPSVSDDQEGGEGPAASGDSGDSQGATRPARSVISSRWHDLDEDLQPAAEAVGPPN<br>DEDMQEAEEGGLVSSGGESPGDSESSDDMLGIIALGKPSMPLACRPVDAFKKEHRISEGTYGVVYKALEKST<br>GRICALKMVKLENEREGFPQTAIREINILLSFHHPNIVNVSEVVVGYSMNHVYMVMDYAEHDLKALQSKQ<br>KKPFTVPQVKYIMQGLLSAMAYLHDNWVLHRDLKTSNILYTNKGEVKICDFGLARQYGSPLRAYTQSVVT<br>MYYRCPEVLIGNLRERTGPNSGRNAKPLSTVPIMYSTALDMWSLGCIMAELLTNKVLFCGKDEYDQLAKIF<br>KLIGLVTEENFPELAKEMPNWKKVEHYFAGIVGGSGSGDFAGLRKMFPPPQVVTSKVPSGPTLTEAGFDLLC<br>GLLRHDPRQRLTAADALEHAWFKETPLPTERVLMPTFAADRQNHL
**CDKH2 Sequence**
**>Bbra:CDKH2 from race A**
MGGSLKRGEKDHPRSSSHDQRRDEGRSERAASAGGGQRRHRPEDEGQRPGGPDTHVHSHHRDRGAYHVP<br>HASSGWRPSGSRLGGPTQSEPGPSKEAGRQAPARPPRSPYGGDSLLSERRGAAAQRLEREQEGRDRGMSGT<br>RQDARGSDSDGNGQDDEEGKLPMNRQDPAPEGGGATVGSAKRRHADEGGEATEERGREGLPPKDVKRV<br>RMASPEVADGRQHSRQHERGGDAARGKGAAAIHTAGSSSRLPAVSPLVSRSAVEIAQQQLELFEQQAQLM<br>EDIDPDAPAAMKPSPSVSDDQEGGEGPAASGDSGDSQGATRPARSVTSSRWHDLDEDMQPAAEAVGSGK<br>DEDMQQAQEGGLVSSWKTSSSDESSDDLFGSDALGRPPLPQACRSVDAFKKMGLISEGTYGVVYKAQEKNS<br>GRICALKMVKMKHEKEGFPLTAIREINILLSFHHPNIVNVSEVLVGNSINKVYMVMDYAEHDLKALQSKQK<br>VPFSVAQVKYIMQGLLSAMAYLHENWVLHRDLKTSNILYTNKGEVKICDFGLARQYGSPLRAYTTPVATLY<br>YRCPELLLGAAMYSTALDMWSLGCIMAELLTNKVLFCGKDERDQLTRIFRLIGPITEDNFPGHAEALPNWK<br>RSGGGYAAIGAGSGDFAGLRKMFPPPPVVSSKVPSGPTLTDAGFDLLCGLLRHDPRNRITAANALEHPWFK<br>ENPLPTERVLMPTFAPDLP
**CDKI1 Sequence**
**>Bbra:CDKI1 from race A**
MAVDTCSGQQVALKRVFKQPHIRGQPPPDENKPLKEVAALRAAHHPNVVGLLDHCIQGDEHILIFELCAV<br>STLEVIREAPQPLDQTLIKTILQSVFQGLEACHTAGVIHRDIKPSNILITHSGNIKLADFGQARSMGVAPDGD<br>QQEETGEKAPPLTAEVATRWYRAPELMYGSTTYGFGVDIWGAGCVFAELLGMAPLFAGISDLDQLNRVVD<br>VLGAPDPVTWPEVVSYPDYGKILIRSTCAPKSLRDLFPDAPSSAVDLLGQCLCANPAGRCSASAALHHPYFC<br>EEPHAAPPEALAAFVREHVPRPGALEGQPELEPFPAARCPCPSCVWGAAT
**MAT3/RBR Sequence**

**>Bbrb:MAT3/RBR from race B (Bobra.0391s0021.1.p)**
MIMADFGAVHFGQLCRSSSLKLDREMLELAETIIEGCKDDVRQRCISSSLPKGHMESFCQAGVCLDALSSKGI<br>VRGEGEGSSGNKTPLLPTLSNILEAFDVRLAPFLKELAVQVATAEPVLPTPWHPDEPLERILKLRELQSNYIGT<br>AVLSKKYRGLYETYCKLPAEETSLPLKLFWTLFLVAKLRLLPQFPDLVTSFGLLIAVFNILLAHLDAQHRRVA<br>HDDNEHFPMRTPSGAADVLESLVRRSKADSSMVKELASKLDDLLVQILPGMAQPASEQASQTQPDGLSTG<br>CIHVEGLLSDPEVTQNVNRMLNESYEAAYGVAEIDERPLLWVDTADLVRSTPPTPSANPAPLSSALASCAMS<br>TPMQYRELTANSPAVPPSALGNNGMNPYIRSGAMRTFASNSSSLITPSPMWNGGRHAYTSDIAETPVKDSN<br>AAVTWLGTVVDAQSQAESSQRASCLGEAGEDVKREVHARINRYAAIVFPEVENIDPYGSTDLKTSGVMRQR<br>TPGVALYHQVLETLLRKDMDRYGREVFLQLVNSVSFHKCLAASAFEVVVASFGMASHTFPTVLERLHMKPF<br>DLCKFIGTFVRNVPGLPKMLKRHMFSIEEKCLESLAWARGSTLYPLLQLACERQEDAVMGDMGSSPGSGSK<br>LALTPTKRTRDGSPIGSVRQTGWPENEDDHVSSSGFHLPGDLGEGMRQSGHGQEGATEPAPLPRAFGSRYN<br>HDDKQPGRQVVFDFLSRVLRLAHIRANDLCERLDFQPLDRSTVIAEVYTMLHYCCFEVTSLLYGRHLDQLLL<br>CAIYGVCKVHQLKQVTFKDIIGQYKKQAQCKTDTFRSVPISLSPDLEIQQTGDVILFYNSVFIPATKQFVLALG<br>QREVPILPQPTLGSNVASRYGTPLRGTLSVLPSPKPAGVGGSLLVSPLRPKVQVSPLGGKPLSVCLGASPHQD<br>VSYLNTILSGICQPKDLPSNLGNAFPPHSSANGHVNVMVDGVVDDYKSKRQGLKSVARDVGLGHVQGVG<br>HNLQPQRGVWPGIES
**CDKA1 Sequence**
**>Bbrb:CDKA1 from race B (KV908774.1) ***
MVPDAPGRRLSGMDQYEKLDKIGEGTYGVVYKARNKQTGQIIALKKIRLEQEEEGVPSTAIREISLLKELQHI<br>NVVRLYDVIHTDKRLHLVFEFCDLDLKKHMDANPHICRDERLVKLYLYQMLQGISYCHSHRVLHRDLKPQ<br>NLLIDRSNNALKLADFGLARAFGLPARQYTHEVVTLWYRAPEILLGAKHYGTPVDIWSIGCIFAEMITQRPL<br>FPGDSEIDELFKIFRILGTPDDTIWQGVSELPDYKDTFPKWQRRPLQEVVPGLNSLGIDLLDKMLRYHPQERIT<br>SKTALNHPYFQDIRVLLTECQYMNNAPTQWAPP
**CDKB1 Sequence**
**>Bbrb:CDKB1 from race B (Bobra.0276s0007.1.p)**
MDQYEKLEKIGEGTYGKVYKARDIQTGKLVALKKTRLEMEQEGVPSTALREVSLLQMLGESNHVVKLLCVE<br>HVEEGGKPVLYLVFEYLNTDLKRYMDRTGKGPNATPLPQNLIKSFMYQLIKGVAHCHKHGVMHRDLKPQ<br>NLLVDDSKKCLKIADLGLGRAFSIPIKSYTHEIVTLWYRAPEVLLGCTHYAPAVDMWSVGCIFAELVRKQAL<br>FPGDSELQQLLHIFKLLGTPDEASWPDVTRLRDWHEFPQWRAQDLQKVFPTLSAEGVALMQRMFTYDPAK<br>RISAKEALKHPYFDDLDKEAVDLLESDIIRNRECF
**CDKC1 Sequence**
**>Bbrb:CDKC1 from race B (Bobra.110_2s0087.1.p)**
MGDTSVGQKRPRASGQNGEVQTSPALNQTRWKVELSSSSSCEEQYGGTRCYETSYQRADQIGEGTYGQVYL<br>ATDLLDGSPVALKKIRMDNEKEGFPITAVREIKLLKMLDHENVIRLREIVRSNGTKDNQFKGSIYMAFDYMD<br>HDLTGLMERQNHKFTLPQIKCYMKQLLQGLNFCHRNGVLHRDLKASNLLINNNGQLKLADFGLARPYRQ<br>SHEGRFTNRVITLWYRPPELLLGSERYGPEIDMWSVGCILAELLLGKPIFPGKDETDQLDLIMKLLGSPNEDN<br>MPGCTKLPHYKLLNHQYKKNKLREHFSKAPSNLVDEHALDLLEKLLCLDPKKRTSALDAIGHSFFWADPQ<br>PCKPEELPRHQPSHEFDMKKLRADQKAAKANAANAGHGSEYHPDKKSRYQAGHHGGRGTGPPQPGGPS<br>YGGPPPQRGPPVQAYQQVGRAPPVPAGGDRHQPPHPHGPGRTHPPQGAGAPPALGPPPPREPYAAGSWD<br>RSSHAAPRHGYDGGGYPMGPSQGGRGGPHPPPRGPPHGPPGHYPPAGPPQRYGAPPAGVPPSGGAPGYPP<br>GRGYTGFAPRGSHGYNQYQRPPGGGNMRDRGGPPPQGQYPRSGSGHQGPPPNWSNQRR
**CDKD1 Sequence**
**>Bbrb:CDKD1 from race B (KV908446.1) ***
MEKYEKGPTLGQGTFGVVFQARNKETGQIVAIKKIRLGEAREGINMTALREIKLLRELESPYIVPLLDVFHHK<br>HNVSLVFEFMESDLEAIIKDRSIVLSASDVKAYMQMALEALAFCHSRWVLHRDIKPNNFLLTATGEMKLAD<br>FGLSRVFGSPDAKYTDQVFSRWYRAPELLFGSTLYGPAVDIWGLGCVFAELLLRKPWLPGNSDIDQLSLIFKA<br>LGTPTDAQWPRMRDLPHFVEFQPMPSPPLRNTFRQASEDALDLLARMMAYDPSRRISAADALKHRYFRAE<br>PAATPPTRLPRPPVKANFPLQVSGVGGSAIQEAVAGEAAAVPMQNGSTGPSANIAGGLAGSEVDGSSGLGD<br>ADGYPPRPKCDVEDLRYLRKRKLFMDEAMHLA

**CDKE1 Sequence**
**>Bbrb:CDKE1 from race B (Bobra.0070s0109.1.p)**
MQNQSAAHVATTPASGAITQAVASYLEAFYDVVGKIGEGTYGVVYLARSREPRPRMLAIKTFKPGKEGDGI<br>SPTAIREITLLRETDHENIIRLDSVNLNRKEASLSLAFDYAEHDLYEMIRFHRDKSQGCQPLDPYTLKSLMWQ<br>LVNGVSYLHQNWIMHRDLKPSNVLVMGDGPEQGCVKIGDFGLARIFKEPLRSLSDNGVVVTIWYRAPELLL<br>GAKHYTTSIDMWAIGCICAELLLLRPLFQGDEKKHPGNAFQADQLDRIFRMLGQPTAKQWAGLEHLHHW<br>RDNTENVRVRRPEHPSSSELAKYLIDNTVVTAGGSSAQPLQASCSLIDLLSRLLDYNPETRLTAFQALEHPYF<br>HEEPLPGRNAFVNNGRVVASYPKRTKYLATAVNMPIHAPNSSYEAPGAPVSTMSRNTTPSSGMMRSTGGL<br>PAGAVRGVPPAHPSGVPRKRKLDQLGPAGFR
**CDKG1 Sequence**
**>Bbrb:CDKG1 from race B (Bobra.0068s0039.1.p)**
MDGKTLPVLSSVTKFEKIARIGEGTYGVVYQARNRQTGEVVALKKVRMDRERDGMPVTSVREMRVLQNCR<br>HPNLVALCEVVTGSKPDSIFLVFEFCEHDLGRLLDSLPNPFTISEVKCLLKQLLEGVAFLHSRWVMHRDIKLS<br>NLLLTRGGHLKLCDFGLARYFRPFQQAYTPRVITLWYRAPEVLLGEDEYTEAVDMWSVGAVFGELLKHEPL<br>FPGKTELDMLSLMASLLGAPSEAIWPGFSKLPGGSSLRLPAQPYNYLAKVFPKVSEAGIDLLNRMLTYDPERR<br>ITARSALKHPFFQEIPLAKAPEDMPTFPAHQSAPAASKRGALRAEADAEDRGLKRVRGGNGLDDRFGEAFG<br>DSVRPVGMRQAYRRA
**CDKH1 Sequence**
**> Bbrb:CDKH1 from race B (Bobra.174_2s0011.1.p)**
MVDARTTVGVEIPTPDGTVRSEIIYEVHRRHDEERVRHREHVGEKSGYAGRTGPRPSALTGSDAPIIADLLRL<br>AKEAADRDRERTLQQAGSQPERTRTKEKGEVSSSDEDPDEEGELPQPHLRAVLSGDVVRAAKRVKISPEGG<br>RWQDREHTSARGIHTAGSSSHLVASPVAGRGASAVGMANAELQQFLHEKPSPLDIDPDAPAAMKPDPSVS<br>DDDRRDDRSSEVSGDSPSANPTRSIVSSKWHNFEEEDPEPAPSQKEQPAGPPQDEAQGGAQQGPASPFDNF<br>GKPHPLIPNRPSFLKPCRSVNDFEKIRRISEGTYGVVYKVRDVTTNKIHALKKVKMEREKDGFPLTSIREINILL<br>SVHHPNIVNVSEVVTGGKEGGDVYMVMDFSEHDLRALQEIKKQPFTVAQVKCIMWQIFSGMAYLHEHWV<br>LHRDLKTSNILYSNKGEVKICDFGLARHYGSPLRPYTQMVVTLYYRAPELLLGATTYSTAVDMWSLGCIMA<br>EVLTNKILFAGKSELEQIQEIYSVIGAPNEDVFPGYKEKLPHLSKLTFKKLPRATVGYENLRAKFPQQPSMFER<br>TTSTAPMLTDAGFDLLKGLLAHDPSKRTKAAQALQHEWFQEAPLPIEPILMPVLPEKGNEPAAKIPKQQAP<br>VIAPYVPRVSSHTASAAMAAAEIRAKFANFAKNR
**CDKI1 Sequence**
**>Bbrb:CDK11 from race B (KV908477.1) ***
MHSRVTCILLMEVGYGDLDLGNEMQDLEIEHGDSDSGGKFENDDAFGGLDDFFGIKRLRSDKHSYVSIGLL<br>GQGGFSDVMVARQEDTGKVVAIKRVFQPFPVRGAPSQELSTRYPELLALISVQHDNVIGLVDHIPDEQSPAL<br>VLEMCASDLWAMIDAAPQPLSQQLMKGVMQGILRGLAACHQAGIIHRDLKPSNVLINYDGQIKLADFGM<br>ALVEDPQADSCGTGVQQERLRTPEVASRWYRAPELLFGSSCYDSAVDIWAAGCIFAEMLGMGPLFAGATDI<br>EQLLRVVEVLGPPHLATWPQLSSLPDYGKIAFAGEGRNKLLEVLPDAPPNAASLLSMFLQYNPVDRPSAAT<br>ALLQPYFTQPPLPASPSSIAAFIRKHVSRPKALQGFSEPFLQGPCPCPSCILDP

* From [57].

Table S3 Putative CDK phosphorylation sites in the retinoblastoma protein sequences (RBR) of *B. braunii* race A (Yamanaka), *Botryococcus braunii* race B (Showa), *Ostreococcus tauri*, *Gonium pectorale*, *Chlamydomonas reinhardtii*, *Volvox carteri* f. *nagariensis* (female), *Volvox carteri* f. *nagariensis* (male) and *Arabidopsis thaliana*.

Species	Phosph. site	S/T-P seq.	Score
1. B. braunii race A (Yamanaka)	570 S	RKAR**SPKK**M	0.998
2. B. braunii race A (Yamanaka)	310 S	RSMMSPIEG	0.997
3. B. braunii race A (Yamanaka)	79 S	QRAGSPDST	0.997
4. B. braunii race A (Yamanaka)	865 S	ELGR**SPRR**S	0.994
5. B. braunii race A (Yamanaka)	562 S	EQPVSPQER	0.989
6. B. braunii race A (Yamanaka)	548 S	DPNPSPPDN	0.988
7. B. braunii race A (Yamanaka)	586 S	GREESPQHF	0.986
8. B. braunii race A (Yamanaka)	454 S	TTIKSPSFH	0.966
9. B. braunii race A (Yamanaka)	952 S	DARGSPIVA	0.942
10. B. braunii race A (Yamanaka)	323 T	RMLLTPSPA	0.926
11. B. braunii race A (Yamanaka)	325 S	LLTP**SPAR**G	0.893
12. B. braunii race A (Yamanaka)	810 T	SRQATPQNS	0.833
13. B. braunii race A (Yamanaka)	263 T	SGAATPSMS	0.795
14. B. braunii race A (Yamanaka)	577 T	KMDL**TPTK**R	0.754
15. B. braunii race A (Yamanaka)	87 T	THRSTPTLS	0.715
16. B. braunii race A (Yamanaka)	343 T	HIMETPVTE	0.710
17. B. braunii race A (Yamanaka)	836 S	SLLV**SPLR**R	0.672
18. B. braunii race A (Yamanaka)	303 T	MLTG**TPMR**S	0.612
19. B. braunii race A (Yamanaka)	402 S	VFPESPQNS	0.600
20. B. braunii race A (Yamanaka)	821 S	AGLASPPPS	0.592
21. B. braunii race A (Yamanaka)	273 T	CNVSTPLQN	0.523
22. B. braunii race A (Yamanaka)	757 T	AIELTPGLQ	0.512
23. B. braunii race A (Yamanaka)	284 S	LTAFSPAIQ	0.501
**1. *B. braunii* race B (Showa)**	659 S	TRDGSPIGS	0.997
**2. *B. braunii* race B (Showa)**	918 S	KVQVSPLGG	0.978
**3. *B. braunii* race B (Showa)**	896 S	SVLPSPKPA	0.977
**4. *B. braunii* race B (Showa)**	427 T	DIAE**TPVK**D	0.960
**5. *B. braunii* race B (Showa)**	505 T	MRQRTPGVA	0.956
**6. *B. braunii* race B (Showa)**	640 S	DMGSSPGSG	0.945
**7. *B. braunii* race B (Showa)**	909 S	SLLV**SPLR**P	0.933
**8. *B. braunii* race B (Showa)**	885 T	SRYG**TPLR**G	0.897
**9. *B. braunii* race B (Showa)**	231 T	FPMRTPSGA	0.805
**10. *B. braunii* race B (Showa)**	344 T	STPPTPSAN	0.722
**11. *B. braunii* race B (Showa)**	123 T	PVLPTPWHP	0.654
**12. *B. braunii* race B (Showa)**	408 T	SSLITPSPM	0.597
**13. *B. braunii* race B (Showa)**	341 T	LVRSTPPTP	0.593
**14. *B. braunii* race B (Showa)**	86 T	SGNKTPLLP	0.587
**15. *B. braunii* race B (Showa)**	410 S	LITPSPMWN	0.583
**16. *B. braunii* race B (Showa)**	650 T	KLAL**TPTK**R	0.580
**17. *B. braunii* race B (Showa)**	363 T	CAMSTPMQY	0.559
**18. *B. braunii* race B (Showa)**	374 S	LTANSPAVP	0.530
**19. *B. braunii* race B (Showa)**	932 S	CLGASPHQD	0.523
**20. *B. braunii* race B (Showa)**	832 S	PISLSPDLE	0.488
1. O. tauri	642 S	KRPVSPMFN	0.996
2. O. tauri	760 S	RLKLSPEVT	0.996
3. O. tauri	698 T	RGAT**TPRR**K	0.995

4. O. tauri	120 S	TRLKSPSLG	0.992
5. O. tauri	371 S	AAPF**SPYR**P	0.991
6. O. tauri	913 S	NIYVSPMRP	0.965
7. O. tauri	479 S	RTDISPHSM	0.953
8. O. tauri	935 T	GEPSTPRTR	0.935
9. O. tauri	302 S	ADVASPVME	0.929
10. O. tauri	973 T	KAT**STPSR**L	0.885
11. O. tauri	390 S	GMSM**SPMR**A	0.833
12. O. tauri	676 S	PQCH**SPVR**R	0.811
13. O. tauri	898 S	FGIS**SPRR**R	0.779
14. O. tauri	691 S	TVFS**SPLR**G	0.769
15. O. tauri	359 T	GSMA**TPTR**R	0.643
16. O. tauri	407 T	VVPPTPISQ	0.624
1. C. reinhardtii	357 S	SPRFSPGHM	0.993
2. C. reinhardtii	840 S	NFDFSPLEG	0.993
3. C. reinhardtii	1010 S	RASQSPRGP	0.993
4. C. reinhardtii	759 S	APPP**SPKR**S	0.991
5. C. reinhardtii	353 S	TKFASPRFS	0.988
6. C. reinhardtii	1066 S	GGAL**SPTK**G	0.957
7. C. reinhardtii	1201 T	RRQR**TPNR**R	0.941
8. C. reinhardtii	447 S	VAEPSPTLQ	0.909
9. C. reinhardtii	732 T	PPSTTPAAP	0.831
10. C. reinhardtii	1078 T	SGHPTPTGP	0.767
11. C. reinhardtii	389 T	LHTATPAHP	0.722
12. C. reinhardtii	467 T	SSGPTPVQQ	0.622
13. C. reinhardtii	773 S	SGMM**SPAK**K	0.568
14. C. reinhardtii	303 S	DLVASPVLE	0.564
15. C. reinhardtii	746 S	PAAASPAAP	0.562
16. C. reinhardtii	423 T	GVPGTPISE	0.555
17. C. reinhardtii	407 S	LGLHSPLPM	0.512
1. G. pectorale	45 S	QERVSPPEF	0.998
2. G. pectorale	662 S	LPPL**SPKR**S	0.998
3. G. pectorale	343 S	SPRYSPGQM	0.992
4. G. pectorale	887 S	SGSL**SPSK**R	0.982
5. G. pectorale	339 S	TKFASPRYS	0.976
6. G. pectorale	985 S	GVAPSPTGD	0.970
7. G. pectorale	963 S	MGGSSPSGE	0.967
8. G. pectorale	394 S	PGLHSPLPG	0.943
9. G. pectorale	409 T	GPPITPVSE	0.889
10. G. pectorale	1020 T	GRQR**TPIR**R	0.841
11. G. pectorale	359 T	RGGS**TPIR**P	0.789
12. G. pectorale	646 T	PASTTPGGI	0.718
13. G. pectorale	674 S	SVWM**SPAK**K	0.624
14. G. pectorale	689 T	APCATPVLD	0.613
15. G. pectorale	389 T	VSAGTPGLH	0.592
16. G. pectorale	1006 S	SLPISPALT	0.592
17. G. pectorale	702 S	TCIGSPASS	0.543
18. G. pectorale	638 T	ASGGTPAAP	0.525
19. G. pectorale	737 S	KFDFSPLGR	0.506
20. G. pectorale	20 T	SLGNTPAAQ	0.503

1. V. carteri f. nagariensis (f)	361 S	SPRFSPRHM	0.996
2. V. carteri f. nagariensis (f)	357 S	TKFASPRFS	0.988
3. V. carteri f. nagariensis (f)	680 S	DTSASPLST	0.974
4. V. carteri f. nagariensis (f)	1099 T	RRQR**TPNR**K	0.953
5. V. carteri f. nagariensis (f)	430 T	GPPVTPVSE	0.898
6. V. carteri f. nagariensis (f)	996 T	LNTC**TPGK**R	0.589
7. V. carteri f. nagariensis (f)	398 S	SYAISPSSM	0.572
8. V. carteri f. nagariensis (f)	937 S	EALRSPGTE	0.554
9. V. carteri f. nagariensis (f)	50 S	QENASPASD	0.547
10. V. carteri f. nagariensis (f)	414 S	ARMHSPLPK	0.544
11. V. carteri f. nagariensis (f)	454 S	AAEPSPSVM	0.520
12. V. carteri f. nagariensis (f)	634 S	WEADSPLYS	0.514
13. V. carteri f. nagariensis (f)	888 S	PTIGSPDNV	0.503
14. V. carteri f. nagariensis (f)	763 S	NFDFSPLQR	0.496
15. V. carteri f. nagariensis (f)	696 S	VLEF**SPVK**K	0.492
1. V. carteri f. nagariensis (m)	796 S	NFDFSPLDR	0.993
2. V. carteri f. nagariensis (m)	715 S	APPP**SPKR**S	0.991
3. V. carteri f. nagariensis (m)	356 S	SPRFSPGHM	0.990
4. V. carteri f. nagariensis (m)	352 S	TKFASPRFS	0.988
5. V. carteri f. nagariensis (m)	393 S	PSSVSPRLN	0.982
6. V. carteri f. nagariensis (m)	50 T	QERATPAEF	0.979
7. V. carteri f. nagariensis (m)	971 S	SANSSPTAV	0.857
8. V. carteri f. nagariensis (m)	728 S	MGSM**SPAK**K	0.848
9. V. carteri f. nagariensis (m)	424 T	GQPSTPVSE	0.797
10. V. carteri f. nagariensis (m)	707 T	LSFSTPLCA	0.629
11. V. carteri f. nagariensis (m)	739 S	GVDGSPQPV	0.567
12. V. carteri f. nagariensis (m)	663 S	APLASPACK	0.522
13. V. carteri f. nagariensis (m)	448 S	AAEPSPGLT	0.521
14. V. carteri f. nagariensis (m)	408 S	AGMHSPLPV	0.497
1. A. thaliana	885 S	QCPGSPKVS	0.995
2. A. thaliana	898 S	VPDM**SPKK**V	0.993
3. A. thaliana	385 S	ISPL**SPHK**S	0.992
4. A. thaliana	685 S	NSFT**SPVK**D	0.991
5. A. thaliana	375 S	DALS**SPAR**T	0.984
6. A. thaliana	942 S	HAYQ**SPSK**D	0.971
7. A. thaliana	665 S	GGIR**SPKR**L	0.970
8. A. thaliana	290 S	KKKPSPASE	0.955
9. A. thaliana	406 T	KLAATPVST	0.841
10. A. thaliana	911 S	NVYV**SPLR**G	0.826
11. A. thaliana	712 S	SAFA**SPTR**P	0.618
12. A. thaliana	9 T	QPPVTPPIE	0.573
13. A. thaliana	423 S	RTVISPLLP	0.560
14. A. thaliana	382 S	RTFISPLSP	0.523
15. A. thaliana	389 S	**SPHK**SPAAK	0.523
16. A. thaliana	430 S	LPKPSPGLE	0.505

Phosph. site, phosphorylated amino acid; S/T-P seq., putative amino acid sequence of phosphorylation site; Score, values above 0.500 indicate positive predictions. The minimum sequence required for a phosphorylation site is S/T-P. A specific consensus phosphorylation site for CDKs is S/T-P-X-K/R where X is any residue. S: Serine, T: Threonine; P: Proline, K: Lysine; R: Arginine; m: male, f: female. Bolds double-underlined are the putative specific consensus phosphorylation sites. The program used was from [65,66,67]. Sequences were from [54]: *B. braunii* race B (Bbrb:MAT3/RBR, Bobra.0391s0021.1.p); *O. tauri* (Ot:MAT3/RBR, Acc. No. OUS45688.1); *C. reinhardtii* (Cr:MAT3/RBR, Acc. No. XP_001696629.1); *G. pectorale* (Gp:MAT3/RBR, Acc. No

BAN18532.1); *V. carteri* f. *nagariensis* female (f) (Vcfn:MAT3/RBR(f), Acc. No. ABM47317.1); *V. carteri* f. *nagariensis* male (m) (Vcfn:MAT3/RBR(m), Acc. No. ADI46925.1); *A. thaliana* (At:RBR1, Acc. No. NP_566417.3).

A domain - RBR

Species/Abbrv	** ** *
1. Bbra:MAT3/RBR	TPVTEAMAAVHWLLHQNMSETQAPEPSMELQRFFSECG--------------EGPGEAIA
2. Bbrb:MAT3/RBR	TPVKDSNAAVTWL--GTVVDAQSQAESSQRASCLGEAG--------------EDVKREVH
3. Ot:MAT3/RBR	TPISQAMASASWL--QDIVCA-----SSDLETKLAELR--------RFVPGEETVIDKLH
4. Cr:MAT3/RBR	TPISEAMGASAWL--RGVTSNMVAEPSPTLQRFLAALP-------INSNSSGPTPVQQLM
5. Gp:MAT3/RBR	TPVSELMSASTWL--RGLATTLTAEPSCTLQRFLAAVP-----VSVAQAGTSLSAAEQLS
6. Vcfn:MAT3/RBR (f)	TPVSEIMSTSAWL--RGITASLAAEPSPSVMRYLAEVAVPPDGEQGACVTVTLSAAEQLN
7. Vcfn:MAT3/RBR (m)	TPVSEVMNISAWL--RGRTANLAAEPSPGLTRYLAAVG----GTRSEYVTGSASTAHQLG
8. At:RBR1	TPVSTAMTTAKWL--RTVISPLLPKPSPGLEHFLKSCD--------------RDITNDVT

Species/Abbrv	*
1. Bbra:MAT3/RBR	AEGV----EQMANLVFPESPQNSQ-DEVG---------VSSISMPNRRQCGIRLYYLVLD
2. Bbrb:MAT3/RBR	HARI----NRYAAIVFPEVENIDP---YG-----STD-LKTSGVMRQRTPGVALYHQVLE
3. Ot:MAT3/RBR	HKKVDVLGQRLGQAIREDALVTTMRTDIS-----PHSMVMDELVRQRTTELVHIFFFFLN
4. Cr:MAT3/RBR	MKRV----RDLVASVIPDEAAPSL---LG-----PFPLLNTSLGAERRIEATKLYCHSLD
5. Gp:MAT3/RBR	SRRI----RELISLVIPDESSSTL---LG-----SFP-LQPRLAAERRMEAYKLYLHSLE
6. Vcfn:MAT3/RBR (f)	NQRV----QELVSCIMPEEKIPSL---LG-----SFPLLQPSLVSERRIEVTKVYYLSLE
7. Vcfn:MAT3/RBR (m)	GQRV----RDLISSIIPEEKIPSL---VG-----AFPLLQPSLTAERRLEVTKLYYHSLD
8. At:RBR1	TRRA----HIILEAIFPNSSLGAQ---CGGGSLQAVDLMDDIWAEQRRLEACKLYYRVLE

Species/Abbrv	* * * * * **
1. Bbra:MAT3/RBR	VLDAMRKGDQEKPGRASFATTIKSPSFHKCMLACAFELVIASYRMVTHTFPTVLERLHLK
2. Bbrb:MAT3/RBR	VLETLLRKDMDRYGREVFLQLVNSVSFHKCLAASAFEVVVASFGMASHTFPTVLERLHMK
3. Ot:MAT3/RBR	FLNRILRAESQVKKNANIVALLQSSRFTKSLLACCMEVIVATYKTSTLTFPATTHLLGIH
4. Cr:MAT3/RBR	SLDTILQNEQRTNGLPAALALLGSAKFQRGLIACCIEVVAACYRMVSCAFPNVLDALRIK
5. Gp:MAT3/RBR	SLETLLVAEEKVNGLAGVVALLSSSKFHRGLVACTVEIVAACYRMVSCAFPKVMDVLHIK
6. Vcfn:MAT3/RBR (f)	SLERILQAEEKNNGVAGVTSLLSAGKFHRALVACSAEVVTACYRMVSCTFPKVLDALHIK
7. Vcfn:MAT3/RBR (m)	SLDNILHTEEKVGGMAGVTSLLSAGKFHRALVACCIEVVAACYRMVSCAFPKVLDALHIK
8. At:RBR1	VLEAMCKAEAQILHANNLNSLLTNERFHRCMLACSAELVLATHKTITMLFPAVLERTGIT

Species/Abbrv	** * ** ** ** * * *
1. Bbra:MAT3/RBR	PFDLCKLISTFVRHLPNLPRELKRHMFSIEEKCLESLSWERGSSLYPLLQTACDPNP---
2. Bbrb:MAT3/RBR	PFDLCKFIGTFVRNVPGLPKMLKRHMFSIEEKCLESLAWARGSTLYPLLQLACE------
3. Ot:MAT3/RBR	PFDLTTIIEPFVRADMDLPREIVRHFNSLEEKTLERLAWCKGSALFDFLQSFQESVN---
4. Cr:MAT3/RBR	AFDMANIIGTFVKSIATLPRELKRHLFLIEEKILECLAWEPGSSLYHLIVNVHTENEAAA
5. Gp:MAT3/RBR	AFDLAKMVQGFVRSVSSLPRELKRHMFLIEEKILESLAWEPGSSLYFHIVNHQSES----
6. Vcfn:MAT3/RBR (f)	AFDLAKMIQCFVRSITTLPRDLKRHLFLVEEKILESLAWEADSPLYSLICST--------
7. Vcfn:MAT3/RBR (m)	AFDLAKMIPSFVKSIETLPRELKRHLFLIEEKIIESLAWEPGSSLYTHIVNISVNHQDIT
8. At:RBR1	AFDLSKVIESFIRHEDSLPRELRRHLNSLEERLLESMVWEKGSSMYNSLIVARPSLALEI

B domain - RBR

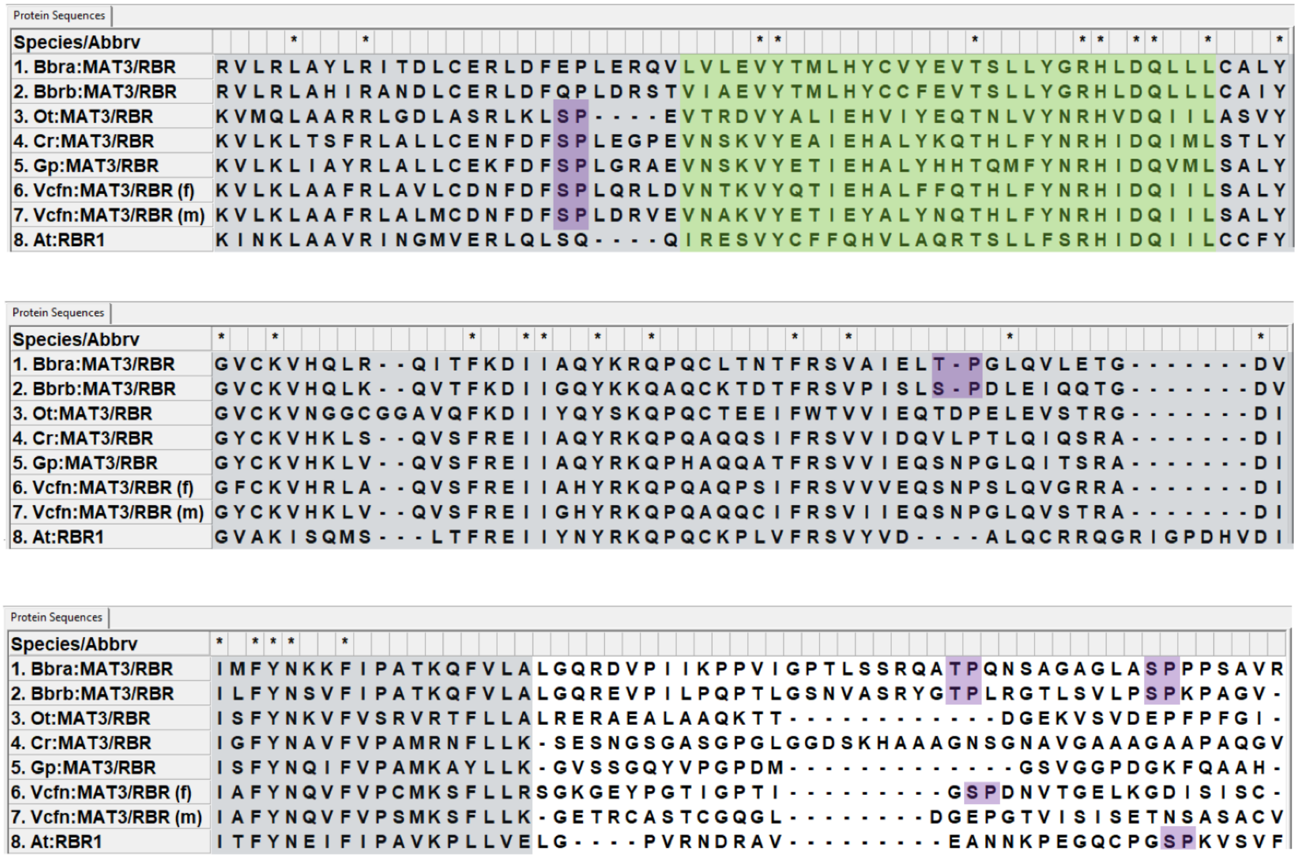

Figure S1. RBR alignment of A and B microalgae domains. (Bbra) and (Bbrb), *B. braunii* race A and B; (Cr), *C. reinhardtii;* (Ot), *O. tauri;* (Gp), *G. pectorale;* (Vcfn (f)), *V. carteri* f. *nagariensis* female; (Vcfn (m)), *V. carteri* f. *nagariensis* male and (At), *A. thaliana* plant. (RBR-A-domain A) and (RBR-B-domain B), gray areas; (LXCXE motif binding site), green area; (phosphorylation sites), violet areas. *B. braunii* race A (Bbra:MAT3/RBR); *B. braunii* race B (Bbrb:MAT3/RBR, Bobra.0391s0021.1.p); *C. reinhardtii* (Cr: MAT3/RBR, Acc. No. XP_001696629.1); *O. tauri* (Ot: MAT3/RBR, Acc. No. OUS45688.1); *G. pectorale* (Gp: MAT3/RBR, Acc. No BAN18532.1); *V. carteri* f. *nagariensis* female (f) (Vcfn: MAT3/RBR (f) Acc. No. ABM47317.1); *V. carteri* f. *nagariensis* male (m) (Vcfn: MAT3/RBR (m), Acc. No. ADI46925.1); *A. thaliana* (At:RBR1, Acc. No. NP_566417.3).

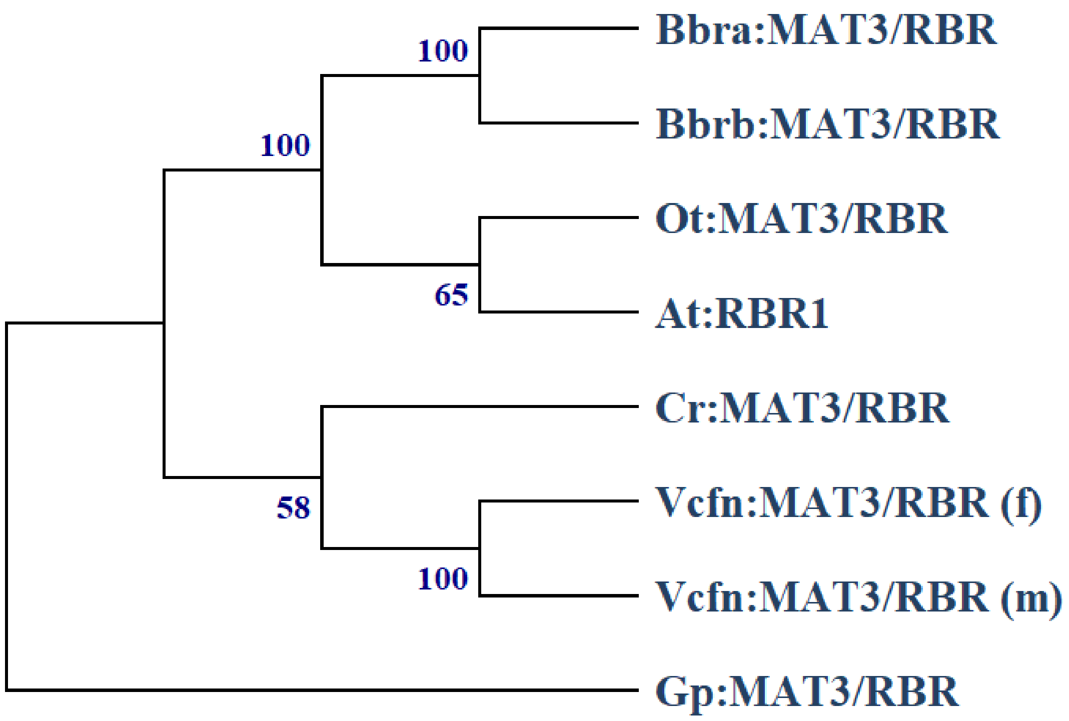

Figure S2. RBR phylogenetic tree of microalgae (Bbra) and (Bbrb), *B. braunii* race A and B; (Cr), *C. reinhardtii;* (Ot), *O. tauri;* (Gp), *G. pectorale;* (Vcfn (f)), *V. carteri* f. *nagariensis* female; (Vcfn (m)), *V. carteri* f. *nagariensis* male and (At), *A. thaliana* plant. *B. braunii* race A (Bbra:MAT3/RBR), *B. braunii* race B (Bbrb:MAT3/RBR, Bobra.0391s0021.1.p), *C. reinhardtii* (Cr:MAT3/RBR, Acc. No. XP_001696629.1), *O. tauri* (Ot:MAT3/RBR, Acc. No. OUS45688.1), *G. pectorale* (Gp:MAT3/RBR, Acc. No BAN18532.1), *V. carteri* f. *nagariensis* female (f) (Vcfn:MAT3/RBR (f), Acc. No. ABM47317.1), *V. carteri* f. *nagariensis* male (m) (Vcfn: MAT3/RBR (m), Acc. No. ADI46925.1), *A. thaliana* (At:RBR1, Acc. No. NP_566417.3).

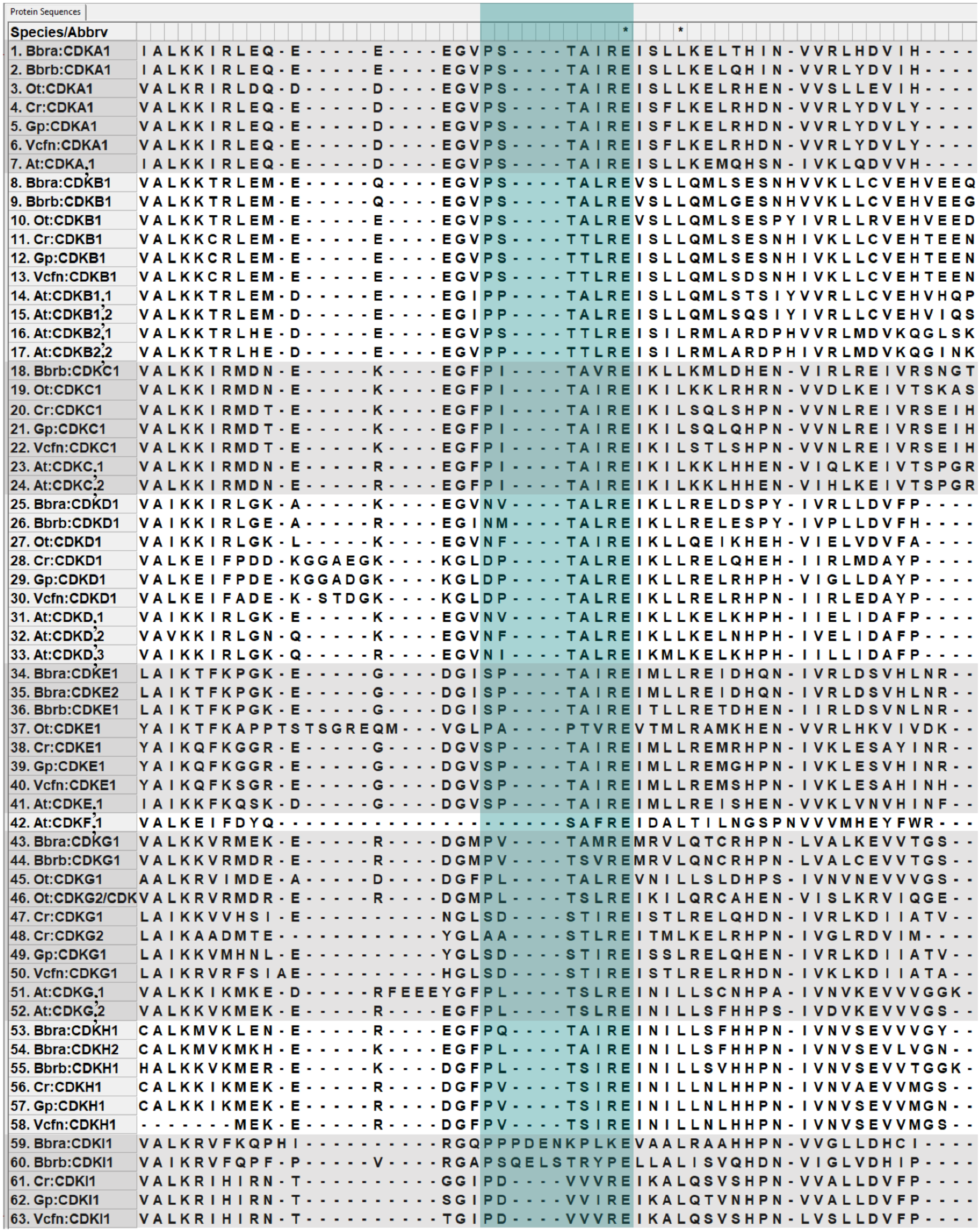

Figure S3. CDKs alignment around PSTAIRE motifs of microalgae. (Bbra) and (Bbrb), *B. braunii* race A and B; (Cr), *C. reinhardtii;* (Ot), *O. tauri;* (Gp), *G. pectorale;* (Vcfn), *V. carteri* f. *nagariensis* and (At), *A. thaliana* plant. The blue shadow region shows the cyclin binding domain. *B. braunii* race A CDKA1 (Bbra:CDKA1), *B. braunii* race A CDKB1 (Bbra:CDKB1), *B. braunii* race A

CDKD1 (Bbra:CDKD1), *B. braunii* race A CDKE1 (Bbra:CDKE1), *B. braunii* race A CDKE2 (Bbra:CDKE2), *B. braunii* race A CDKG1 (Bbra:CDKG1), *B. braunii* race A CDKH1 (Bbra:CDKH1), *B. braunii* race A CDKH2 (Bbra:CDKH2), *B. braunii* race A CDKI1 (Bbra:CDKI1). *B. braunii* race B CDKA1 (Bbrb:CDKA1, KV908774.1), *B. braunii* race B CDKB1 (Bbrb:CDKB1, Bobra.0276s0007.1.p), *B. braunii* race B CDKC1 (Bbrb:CDKC1, Bobra.110_2s0087.1.p), *B. braunii* race B CDKD1 (Bbrb:CDKD1, KV908446.1), *B. braunii* race B CDKE1 (Bbrb:CDKE1, Bobra.0070s0109.1.p), *B. braunii* race B CDKG1 (Bbrb:CDKG1, Bobra.0068s0039.1.p), *B. braunii* race B CDKH1 (Bbrb:CDKH1, Bobra.174_2s0011.1.p), *B. braunii* race B CDKI1 (Bbrb:CDKI1, KV908477.1). *C. reinhardtii* CDKA1 (Cr:CDKA1, Acc. No. XP_001698637.1), *C. reinhardtii* CDKB1 (Cr:CDKB1, Acc. No. XP_001701299.1), *C. reinhardtii* CDKC1 (Cr:CDKC1, Acc. No. XP_001694199.1), *C. reinhardtii* CDKD1 (Cr:CDKD1, Acc. No. XP_001694537.1), *C. reinhardtii* CDKE1 (Cr:CDKE1, Acc. No. PNW83964.1), *C. reinhardtii* CDKG1 (Cr:CDKG1, Acc. No. XP_001696492.1), *C. reinhardtii* CDKG2 (Cr:CDKG2, Acc. No. XP_001701126.1), *C. reinhardtii* CDKH1 (Cr:CDKH1, Acc. No. XP_001702056.1), *C. reinhardtii* CDKI1 (Cr:CDKI1, Acc. No. XP_001700559.1). *O. tauri* CDKA1 (Ot:CDKA1, Acc. No. XP_003078530.1), *O. tauri* CDKB1 (Ot:CDKB1, Acc. No. XP_003083211.1), *O. tauri* CDKC1 (Ot:CDKC1, Acc. No. AAV68597.1), *O. tauri* CDKD1 (Ot:CDKD1, Acc. No. AAV68598.1), *O. tauri* CDKE1 (Ot:CDKE1, Acc. XP_022840036.1), *O. tauri* CDKG1 (Ot: CDKG1, Acc. XP_003074327.1), *O. tauri* CDKG2/CDK10 (Ot:CDKG2/CDK10, Acc. XP_003080520.2). *G. pectorale* CDKA1 (Gp:CDKA1, Acc. No. KXZ46110.1), *G. pectorale* CDKB1 (Gp:CDKB1, Acc. No. KXZ43845.1), *G. pectorale* CDKC1 (Gp:CDKC1, Acc. No. KXZ43562.1), *G. pectorale* CDKD1 (Gp:CDKD1, Acc. No. KXZ54250.1), *G. pectorale* CDKE1 (Gp:CDKE1, Acc. No. KXZ45461.1), *G. pectorale* CDKG1 (Gp:CDKG1, Acc. No. KXZ52035.1), *G. pectorale* CDKH1 (Gp:CDKH1, Acc. No. KXZ53820.1), *G. pectorale* CDKI1 (Gp:CDKI1, Acc. No. KXZ49794.1). *V. carteri* f. *nagariensis* CDKA1 (Vcfn:CDKA1, Acc. No. XP_002949867.1), *V. carteri* f. *nagariensis* CDKB (Vcfn:CDKB1, Acc. No. XP_002947156.1), *V. carteri* f. *nagariensis* CDKC1 (Vcfn:CDKC1, Acc. No. XP_002954450.1), *V. carteri* f. *nagariensis* CDKD1 (Vcfn:CDKD1, Acc. No. XP_002954735.1), *V. carteri* f. *nagariensis* CDKE1 (Vcfn:CDKE1, Acc. No. XP_002957533.1), *V. carteri* f. *nagariensis* CDKG1 (Vcfn:CDKG1, Acc. No. XP_002946192.1), *V. carteri* f. *nagariensis* CDKH1 (Vcfn:CDKH1, Acc. No. - XP_002956993.1), *V. carteri* f. *nagariensis* CDKI1 (Vcfn:CDKI1, Acc. No. XP_002956880.1). *A. thaliana* CDKA;1 (At:CDKA;1, Acc. No. NP_566911.1), *A. thaliana* CDKB1;1 (At:CDKB1;1, Acc. No. NP_190986.1), *A. thaliana* CDKB1;2 (At:CDKB1;2, Acc. No. NP_001031507.1), *A. thaliana* CDK2;1 (At:CDKB2;1, Acc. No. NP_177780.1), *A. thaliana* CDKB2;2 (At:CDKB2;2, Acc. No. NP_173517.1), *A. thaliana* CDKC;1 (At:CDKC;1, Acc. No. NP_196589.1), *A. thaliana* CDKC;2 (At:CDKC;2, Acc. No. NP_201301.1), *A. thaliana* CDKD;1 (At:CDKD;1, Acc. No. NP_177510.1), *A. thaliana* CDKD;2 (At:CDKD;2, Acc. No. NP_176847.1), *A. thaliana* CDKD;3 (At:CDKD;3, Acc. No. NP_173244.1), *A. thaliana* CDKE;1 (At:CDKE;1, Acc. No. NP_201166.1), *A. thaliana* CDKF;1 (At:CDKF;1, Acc. No. NP_001329562.1), *A. thaliana* CDKG;1 (At:CDKG;1, Acc. No. OAO92015.1), *A. thaliana* CDKG;2 (At:CDKG;2, NP_001154456.1).
